# Implant Selection Patterns and Associated Clinical Determinants in Full-Mouth Rehabilitation: A Retrospective Cohort Analysis

**DOI:** 10.7759/cureus.112304

**Published:** 2026-07-08

**Authors:** Jay Gohil, M.G. Dharmendra Kumar, Prachi Dwivedi, Nishtha Agrawal, Shilpa Duseja, Tushar Ranjan

**Affiliations:** 1 Department of Prosthodontics, K. M. Shah Dental College and Hospital, Sumandeep Vidyapeeth (Deemed to be University), Vadodara, IND; 2 Department of Oral and Maxillofacial Surgery, CKS Theja Institute of Dental Sciences and Research, Tirupati, IND; 3 Department of Oral and Maxillofacial Surgery, Government District Hospital, Shivpuri, IND; 4 Department of Prosthodontics, Government College of Dentistry, Indore, IND; 5 Department of Periodontology, Narsinhbhai Patel Dental College and Hospital, Visnagar, IND; 6 Department of Prosthodontics, Buddha Institute of Dental Sciences and Hospital, Patna, IND

**Keywords:** dental implants, full-mouth rehabilitation, generalized estimating equations, implant diameter, implant selection

## Abstract

Introduction: Implant-supported full-mouth rehabilitation is a predictable treatment modality for managing edentulous patients. Appropriate implant selection, particularly implant diameter, is influenced by several patient-, site-, implant-, and prosthetic-related factors. However, limited evidence is available regarding the clinical determinants that guide implant selection patterns in routine full-mouth rehabilitation. This study aimed to evaluate implant selection patterns in full-mouth rehabilitation and identify factors associated with the selection of wide-diameter implants. Materials and methods: This retrospective study reviewed the records of patients who underwent implant-supported full-mouth rehabilitation between January 2016 and December 2022. Data on patient demographics, smoking status, systemic health, jaw location, implant region, bone quality, bone augmentation, implant dimensions, implant characteristics, prosthetic design, and loading protocol were collected. The primary outcome was the selection of wide-diameter implants (≥4.5 mm). Descriptive statistics were calculated, and associations between variables and implant diameter categories were assessed using chi-square and independent-samples t-tests. Generalized estimating equations (GEE) were used to identify independent predictors while accounting for the clustering of arches within patients. Statistical significance was set at p < 0.05. Results: A total of 178 patients contributed to 250 rehabilitated arches. Wide-diameter implants were selected for 43 (17.2%) arches, whereas non-wide-diameter implants were used for 207 (82.8%) arches. Wide-diameter implant selection was significantly associated with mandibular arches (p = 0.004), posterior implant regions (p = 0.001), poor bone quality (p < 0.001), bone augmentation procedures (p < 0.001), greater implant length (12.3 ± 2.1 mm; p = 0.002), fixed full-arch prostheses (p = 0.018), and loading protocols (p = 0.012). Multivariable GEE analysis identified bone augmentation (adjusted odds ratio (aOR) = 3.12, p < 0.001), poor bone quality (aOR = 2.64, p < 0.001), posterior implant region (aOR = 2.18, p < 0.001), mandibular arch (aOR = 1.87, p = 0.010), smoking (aOR = 1.88, p = 0.024), fixed full-arch prosthesis (aOR = 1.74, p = 0.039), and increasing age (aOR = 1.03, p = 0.046) as independent predictors of wide-diameter implant selection. Conclusions: Implant selection for full-mouth rehabilitation is influenced by multiple anatomical, biological, and prosthetic factors. Bone augmentation, poor bone quality, posterior implant location, mandibular arch placement, smoking status, fixed full-arch prosthetic design, and increasing age were independently associated with wide-diameter implant selection, highlighting the importance of individualized treatment planning for implant-supported rehabilitation.

## Introduction

Implant-supported full-mouth rehabilitation has become a widely accepted treatment modality for the restoration of completely or partially edentulous patients, offering improved function, esthetics, comfort, and quality of life compared with conventional prosthetic options [1]. The long-term success of implant therapy depends on numerous factors, including patient-related characteristics, anatomical site conditions, implant design features, and prosthetic treatment protocols [2]. Appropriate implant selection, particularly with respect to implant diameter, length, surface characteristics, and connection type, plays a crucial role in achieving optimal primary stability, favorable load distribution, peri-implant tissue health, and long-term treatment outcomes [2,3].

The selection of implant dimensions and design characteristics is influenced by several clinical considerations, including bone quality and quantity, arch location, available restorative space, loading protocol, and the need for adjunctive procedures, such as bone augmentation [4,5]. In full-mouth rehabilitation cases, clinicians are often required to balance biomechanical demands with anatomical limitations to ensure predictable treatment success. Despite the growing popularity of implant-supported full-arch rehabilitation, considerable variation in implant selection patterns persists among clinicians and treatment centers. Understanding the factors that influence implant selection can provide useful insights into current clinical practices and help optimize treatment planning strategies [6].

Previous studies have primarily focused on implant survival rates, prosthetic outcomes, and patient satisfaction. In contrast, relatively little attention has been paid to identifying the determinants of implant selection in routine clinical practice [7,8]. Retrospective analyses of clinical records offer an opportunity to evaluate real-world treatment patterns and to explore associations among patient characteristics, site-related factors, prosthetic considerations, and implant selection decisions. This study aimed to evaluate implant selection patterns in implant-supported full-mouth rehabilitation and to identify the clinical determinants associated with the selection of wide-diameter implants. The primary objective was to identify independent patient-related, site-related, implant-related, and prosthetic-related factors associated with the selection of wide-diameter implants using multivariable generalized estimating equations (GEE). The secondary objectives were to describe implant selection characteristics, including implant diameter, length, surface characteristics, implant-abutment connection design, prosthetic design, and loading protocol, and to evaluate the associations between demographic, anatomical, and prosthetic variables and implant diameter selection.

## Materials and methods

Study design and setting

This retrospective study was conducted at the Department of Prosthodontics, Buddha Institute of Dental Sciences and Hospital, Bodhgaya, India. This study involved a comprehensive review of clinical records, radiographic data, implant treatment plans, and prosthetic rehabilitation records of patients who underwent implant-supported full-mouth rehabilitation between January 2016 and December 2022. This study was designed to evaluate implant selection patterns and identify factors associated with the clinical choice of implant dimensions and characteristics in full-mouth rehabilitation cases.

Ethical considerations

The study protocol was reviewed and approved by the Institutional Ethical Committee of the Buddha Institute of Dental Sciences and Hospital (approval number: EC/NEW/INST/2023/4016/2023/014, dated February 15, 2023) prior to data collection. As the investigation involved a retrospective analysis of existing patient records without any direct patient intervention, the requirement for obtaining individual informed consent was waived in accordance with institutional ethical guidelines. All patient information was anonymized before data extraction and analysis to ensure confidentiality.

Sample size calculation

Sample size estimation was performed a priori using G*Power software version 3.1.9.7 (Heinrich Heine University Düsseldorf, Düsseldorf, Germany). The calculation was based on multivariable logistic regression analysis, assuming ten independent predictor variables, an anticipated odds ratio of 1.5 for the primary outcome of wide-diameter implant selection, a two-tailed alpha level of 0.05, and a statistical power of 80%. The minimum required sample size was estimated to be 243. To compensate for potentially incomplete records, the final target sample size was increased to 250 arches. The adequacy of the sample was further verified using the events per variable criterion, ensuring a minimum of ten outcome events per predictor variable included in the regression model [9].

Study population and sampling

The study population comprised patients who underwent implant-supported full-mouth rehabilitation during the study period. Consecutive eligible records were screened using the departmental electronic database and archived patient files. A census sampling approach was employed, in which all records meeting the eligibility criteria during the study period were included (Table 1).

**Table 1:** Inclusion and exclusion criteria for study enrollment

Criterion type	Requirement	Description/rationale
Inclusion criteria	Age	Patients aged 18 years or older.
	Treatment type	Underwent implant-supported rehabilitation of one or both completely edentulous arches.
	Record completeness	Complete clinical, radiographic, surgical, and prosthetic records available for review.
	Parameter documentation	Detailed documentation of implant selection parameters, specifically including implant diameter, implant length, connection design, and prosthetic loading protocol.
Exclusion criteria	Incomplete data	Records with incomplete documentation (missing any of the required clinical, radiographic, surgical, or prosthetic data).
	Pre‑prosthetic failure	Patients experiencing implant failure before prosthetic loading (i.e., failure prior to final restoration delivery).
	Specialized implant systems	Cases involving non‑standard implant types, including zygomatic and pterygoid implants, as well as other specialized implant systems.
	Anatomical or syndromic anomalies	Patients with craniofacial syndromes or major maxillofacial defects requiring customized rehabilitation (outside standard prosthetic protocols).

Data collection procedure

**Figure 1:**
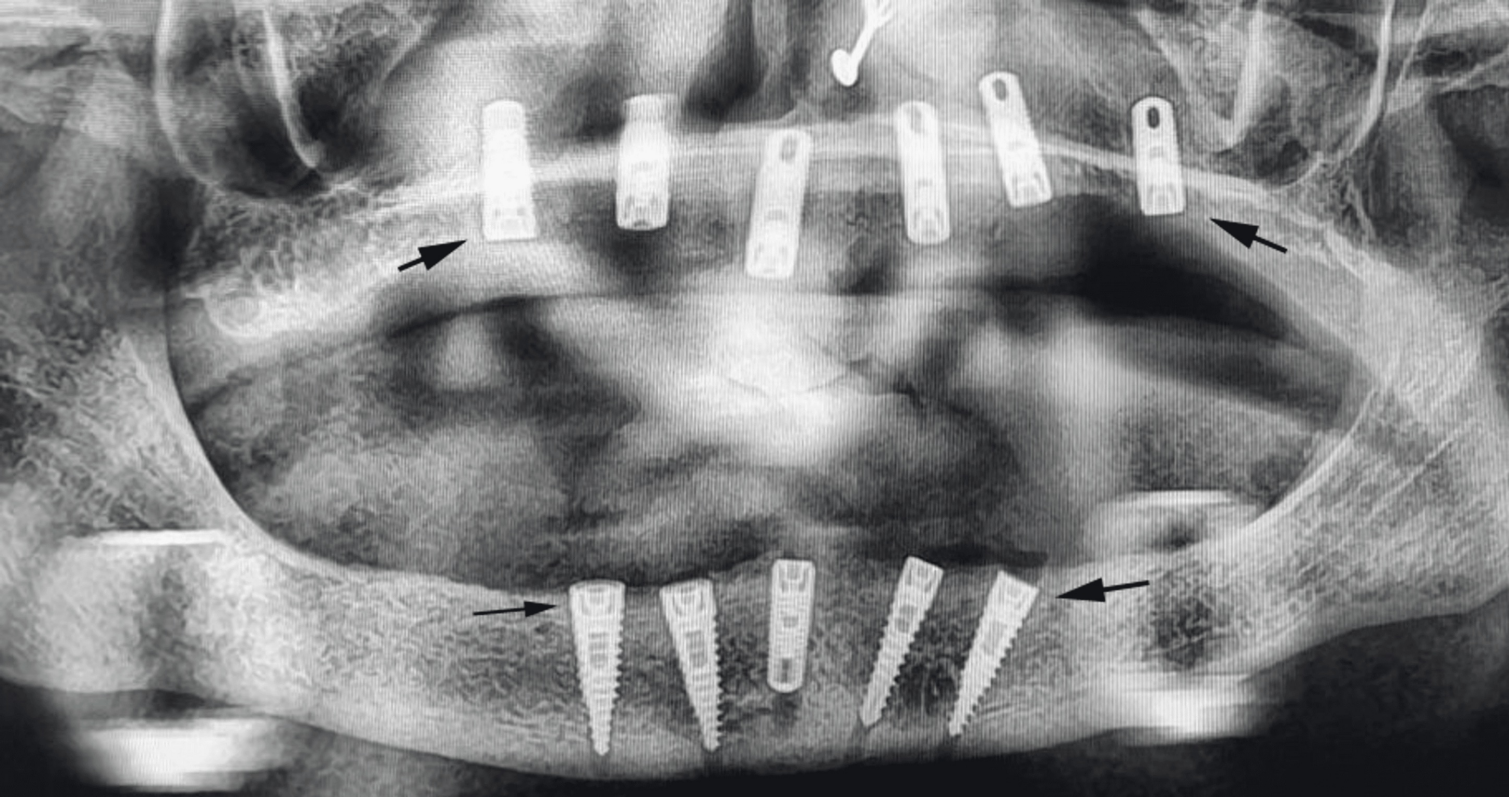
Orthopantomogram showing edentulous maxillary and mandibular jaw with multiple implants (black arrows) placed for full-mouth rehabilitation Original image from study data.

All implant-supported full-mouth rehabilitation procedures included in the study were performed by a single experienced prosthodontist (TR) in accordance with standardized surgical and prosthetic protocols. Rehabilitation involved the maxillary arch, mandibular arch, or both arches depending on the individual treatment plan and extent of edentulism (Figure 1). Implant positions were determined using prosthetically driven planning based on clinical examination and radiographic findings.

The use of a single operator helped minimize variations in implant selection and treatment approach, thereby improving consistency across cases included in the analysis. Patient records were independently reviewed by two calibrated investigators (JG and PD). Prior to formal data collection, a pilot evaluation of 20 randomly selected records was performed to standardize the data extraction procedures and minimize observer variability. Any discrepancies between the investigators were resolved through discussion and consensus with another prosthodontist (NA). Data were extracted from clinical examination records, cone-beam computed tomography reports, panoramic radiographs, surgical notes, implant placement records, and prosthetic treatment documentation. All collected information was entered into a dedicated electronic database for statistical analysis.

Variables assessed

Patient-related variables included age at implant placement, sex, smoking status, and the presence of systemic diseases, such as diabetes mellitus, hypertension, or other medically relevant conditions. Site-related variables included the rehabilitated arch (maxilla or mandible), implant region (anterior or posterior), bone quality, and requirement for bone augmentation procedures. Bone quality was classified as Types I, II, III, and IV based on radiographic and surgical assessments [10].

The implant-related variables included implant diameter, length, surface characteristics, and implant-abutment connection design. Implant diameter was categorized as non-wide diameter (<4.5 mm) or wide diameter (≥4.5 mm). The implant length was recorded in millimeters in accordance with the manufacturer’s specifications [11]. The prosthetic variables included the type of prosthesis, loading protocol, and rehabilitation design. The prostheses were categorized as fixed full-arch prostheses (all-on-4/6 concept), implant-supported overdentures, or implant-supported fixed partial prostheses [12]. Loading protocols were classified as immediate loading (within 48 hours), early loading (one week to two months), and conventional loading (more than two months after implant placement) [13].

Outcome measures

The primary outcome variable was the implant selection pattern, with particular emphasis on the implant diameter selection. Wide-diameter implant selection was defined as the predominant use of implants ≥4.5 mm in diameter within the rehabilitated arch. The secondary outcomes included implant length selection, connection type, and surface characteristics used during full-mouth rehabilitation. Data were initially entered into Microsoft Excel (Microsoft Corporation, Redmond, WA, USA) and subsequently verified using double-entry validation. A random 10% cross-check of the records was performed to identify transcription errors and ensure data integrity. Missing or inconsistent information was verified against the original patient files before the final dataset was locked.

Statistical analysis

Statistical analysis was performed using SPSS Statistics version 26.0 (IBM Corp. Released 2018. IBM SPSS Statistics for Windows, Version 26.0. Armonk, NY: IBM Corp.) by a blinded statistician (MGDK). The distribution of continuous variables was assessed using the Shapiro-Wilk test. Normally distributed continuous variables were expressed as mean ± standard deviation (SD), whereas categorical variables were summarized as frequencies and percentages.

Patient-level characteristics were summarized at the individual patient level, and implant- and site-related variables were analyzed at the arch level. As some patients contributed data from both arches, resulting in correlated observations, GEE were employed to account for within-patient clustering and repeated measures. For the univariable analysis, associations between categorical variables and implant diameter categories were assessed using the chi-square test, with effect sizes quantified using Cramér's V to measure the strength of these associations. Continuous variables were compared using the independent-samples t-test, and their effect sizes were calculated as Cohen's d. Variables with a significance level of p < 0.20 during the univariable analysis were considered eligible for multivariable modeling.

Multivariable analysis was performed using a GEE logistic regression model with a binomial distribution and a logit link function. Adjusted odds ratios (aORs) with corresponding 95% confidence intervals (CIs) were calculated using robust sandwich standard errors. Model fit was evaluated using the quasi-likelihood under the independence model criterion (QIC), and the working correlation structure with the lowest QIC value was selected for the final model. Statistical significance was set at p < 0.05 using two-tailed tests throughout the analysis.

## Results

Patient-level demographic characteristics

A total of 178 patients were included in the study, contributing 250 rehabilitated edentulous arches for the analysis. The mean age of the study population was 54.1 ± 11.2 years. Of the 178 patients, 138 (77.5%) were males, and 40 (22.5%) were females. Full-mouth rehabilitation involving both arches was performed in 72 (40.4%) patients, whereas 106 (59.6%) patients underwent rehabilitation of a single arch. Regarding smoking status, 127 (71.3%) patients were non-smokers, 33 (18.5%) were current smokers, and 18 (10.2%) were former smokers. Systemic diseases were present in 62 (34.8%) patients, whereas 116 (65.2%) had no reported systemic condition. Among the medically compromised patients, diabetes mellitus was observed in 30 (16.9%) patients, hypertension in 21 (11.8%) patients, and other systemic conditions in 11 (6.1%) patients (Table 2).

**Table 2:** Demographic characteristics of study participants (patient-level characteristics) Continuous data expressed as mean ± SD, categorical data as n (%). SD: standard deviation

Patient-level characteristics		Overall, n = 178
Age (years)	Mean ± SD	54.1 ± 11.2
Sex, n (%)	Male	138 (77.5)
	Female	40 (22.5)
Full arch rehabilitation, n (%)	Both arches	72 (40.4)
	Single arch	106 (59.6)
Smoking status, n (%)	Non-smoker	127 (71.3)
	Active smoker	33 (18.5)
	Former smoker	18 (10.2)
Systemic disease, n (%)	None	116 (65.2)
	Diabetes mellitus	30 (16.9)
	Hypertension	21 (11.8)
	Other	11 (6.1)

Arch-level site-related and implant characteristics

Among the 250 rehabilitated arches, 118 (47.2%) were maxillary, and 132 (52.8%) were mandibular. Wide-diameter implants (≥4.5 mm) were selected for 43 (17.2%) arches, whereas non-wide-diameter implants (<4.5 mm) were selected for 207 (82.8%) arches. With respect to jaw location, 26 (60.5%) of the 43 wide-diameter arches were located in the mandible compared with 17 (39.5%) in the maxilla, demonstrating a significant association between mandibular arches and wide-diameter implant selection (p = 0.004). Regarding the implant region, 31 (72.1%) wide-diameter arches were located in the posterior regions, whereas only 12 (27.9%) were located in the anterior regions (p = 0.001). Bone quality was significantly associated with implant diameter selection (p < 0.001). Among the 43 wide-diameter arches, 18 (41.9%) exhibited Type II bone quality, 17 (39.5%) exhibited Type III bone quality, 4 (9.3%) exhibited Type I bone quality, and 4 (9.3%) exhibited Type IV bone quality. Bone augmentation procedures were required in 87 (34.8%) arches, including 12 (27.9%) wide-diameter arches and 75 (36.2%) non-wide-diameter arches, demonstrating a significant association with implant diameter category (p < 0.001) (Table 3).

**Table 3:** Association of site-related, implant-related, and prosthetic factors with implant diameter category Data are presented as mean ± SD or n (%). The chi-square test was used for categorical variables. The independent-samples t-test was used for continuous variables. * p < 0.05 was considered statistically significant; wide-diameter implants were defined as ≥4.5 mm and non-wide-diameter implants as <4.5 mm. SLA: sandblasted large-grit acid-etched, SD: standard deviation, N: number of arches, df: degrees of freedom, effect sizes: Cramér's V for categorical variables and Cohen's d for continuous variables

Variable		Overall (N = 250)	Non-wider (<4.5 mm)	Wider diameter (≥4.5 mm)	Test value (df)	p-value	Effect size
Jaw location, n (%)	Maxilla	118 (47.2)	101 (48.8)	17 (39.5)	9.68 (1)	0.004*	0.19
	Mandible	132 (52.8)	106 (51.2)	26 (60.5)			
Region, n (%)	Anterior	82 (32.8)	70 (33.8)	12 (27.9)	17.62 (1)	0.001*	0.26
	Posterior	168 (67.2)	137 (66.2)	31 (72.1)			
Bone quality, n (%)	Type I	46 (18.4)	42 (20.3)	4 (9.3)	22.44 (3)	<0.001*	0.30
	Type II	118 (47.2)	100 (48.3)	18 (41.9)			
	Type III	73 (29.2)	56 (27.1)	17 (39.5)			
	Type IV	13 (5.2)	9 (4.3)	4 (9.3)			
Bone augmentation performed, n (%)	Yes	87 (34.8)	75 (36.2)	12 (27.9)	25.87 (1)	<0.001*	0.32
	No	163 (65.2)	132 (63.8)	31 (72.1)			
Implant length, n (%)	8 mm	15 (6.0)	14 (6.8%)	1 (2.3)	11.72 (4)	0.024	0.21
	10 mm	50 (20.0)	45 (21.7%)	5 (11.6)			
	11.5 mm	90 (36.0)	77 (37.2%)	13 (30.2)			
	13 mm	68 (27.2)	55 (26.6%)	13 (30.2)			
	15 mm	27 (10.8)	16 (7.7%)	11 (25.6)			
Implant length (mm)	Mean ± SD	11.4 ± 1.9	11.2 ± 1.6	12.3 ± 2.1	3.16 (248)	0.002*	0.53
Implant surface, n (%)	SLA (sandblasted)	148 (59.2)	126 (60.9%)	22 (51.2)	0.27 (3)	0.602	0.03
	Oxidized/TiUnite	61 (24.4)	49 (23.7%)	12 (27.9)			
	Hydrophilic SLA (SLActive)	29 (11.6)	24 (11.6%)	5 (11.6)			
	Other/machined	12 (4.8)	8 (3.9%)	4 (9.3)			
Connection type, n (%)	Internal hex	142 (56.8)	119 (57.5%)	23 (53.5)	0.18 (2)	0.669	0.02
	External hex	63 (25.2)	52 (25.1%)	11 (25.6)			
	Morse taper/conical	45 (18.0)	36 (17.4%)	9 (20.9)			
Type of prosthesis, n (%)	Fixed full-arch (all-on-4/6)	112 (44.8)	88 (42.5%)	24 (55.8)	4.87 (2)	0.018*	0.14
	Implant-supported overdenture	78 (31.2)	67 (32.4%)	11 (25.6)			
	Implant-supported fixed partial prosthesis	60 (24.0)	52 (25.1%)	8 (18.6)			
Loading protocol, n (%)	Immediate (within 48 hours)	56 (22.4)	47 (22.7%)	9 (20.9)	5.01 (1)	0.012*	0.14
	Early (1 week to 2 months)	62 (24.8)	53 (25.6%)	9 (20.9)			
	Conventional (>2 months)	132 (52.8)	107 (51.7%)	25 (58.1)			

The mean implant length across all arches was 11.4 ± 1.9 mm. Arches restored with wide-diameter implants demonstrated a significantly greater mean implant length (12.3 ± 2.1 mm) than non-wide-diameter arches (11.2 ± 1.6 mm) (p = 0.002). Regarding implant surface characteristics, sandblasted large-grit acid-etched (SLA) surfaces were the most frequently used, accounting for 148 (59.2%) arches, followed by oxidized/TiUnite surfaces in 61 (24.4%) arches, hydrophilic SLA surfaces in 29 (11.6%) arches, and other or machined surfaces in 12 (4.8%) arches. No significant association was observed between the implant surface characteristics and implant diameter category (p = 0.602). Similarly, the implant-abutment connection type did not significantly influence the implant diameter selection (p = 0.669), with internal hex connections used in 142 (56.8%) arches, external hex connections in 63 (25.2%) arches, and Morse taper/conical connections in 45 (18.0%) arches (Table 3).

Prosthetic factors and loading protocol

Fixed full-arch prostheses based on the all-on-4/6 concept were the most common rehabilitation modality, accounting for 112 arches (44.8%), followed by implant-supported overdentures in 78 arches (31.2%) and implant-supported fixed partial prostheses in 60 arches (24.0%). Among the 43 arches restored predominantly with wide-diameter implants, 24 (55.8%) received fixed full-arch prostheses, 11 (25.6%) received implant-supported overdentures, and 8 (18.6%) received fixed-partial prostheses. The type of prosthesis was significantly associated with implant diameter selection (p = 0.018) (Table 3). Conventional loading was employed in 132 (52.8%) arches, early loading in 62 (24.8%) arches, and immediate loading in 56 (22.4%) arches. Among the wide-diameter arches, 25 (58.1%) underwent conventional loading, 9 (20.9%) underwent early loading, and 9 (20.9%) underwent immediate loading. The loading protocol was significantly associated with implant diameter selection in the univariable analysis (p = 0.012) (Table 3).

Multivariable GEE regression analysis

The GEE model identified several independent predictors of wide-diameter implant selection after accounting for the clustering of arches within patients (Table 4). Bone augmentation emerged as the strongest predictor of wide-diameter implant selection (adjusted OR = 3.12; 95% CI: 1.89-5.16; p < 0.001). Arches with poorer bone quality (Types III/IV) were 2.64 times more likely to receive wide-diameter implants than arches with better bone quality (adjusted OR = 2.64; 95% CI: 1.65-4.23; p < 0.001). Posterior implant placement was associated with a 2.18-fold increase in the likelihood of wide-diameter implant selection (adjusted OR = 2.18; 95% CI: 1.41-3.36; p < 0.001), and mandibular arches were 1.87 times more likely to receive wide-diameter implants than the maxillary arches (adjusted OR = 1.87; 95% CI: 1.16-3.01; p = 0.010).

**Table 4:** Multivariable GEE analysis of factors associated with wide-diameter implant selection The outcome variable was wide-diameter implant selection (≥4.5 mm). GEE logistic regression with robust standard errors and an exchangeable correlation structure was used; data are presented as β coefficient, SE, aOR, and 95% CI. Wald chi-square test was used to assess statistical significance. * p < 0.05 was considered statistically significant. aOR: adjusted odds ratio, CI: confidence interval, SE: standard error, GEE: generalized estimating equations, QIC: quasi-likelihood under the independence model criterion

Predictor variable	β coefficient	Robust SE	aOR (95% CI)	Wald χ²	p-value
Age (per year increment)	0.030	0.015	1.03 (1.00-1.06)	4.00	0.046*
Male sex	0.351	0.263	1.42 (0.85-2.38)	1.78	0.182
Posterior implant region	0.780	0.221	2.18 (1.41-3.36)	12.46	<0.001*
Mandibular arch	0.626	0.243	1.87 (1.16-3.01)	6.64	0.010*
Bone quality (Type III/IV)	0.971	0.240	2.64 (1.65-4.23)	16.37	<0.001*
Bone augmentation performed	1.139	0.256	3.12 (1.89-5.16)	19.80	<0.001*
Systemic disease present	0.444	0.285	1.56 (0.89-2.73)	2.43	0.119
Smoking	0.632	0.279	1.88 (1.09-3.25)	5.13	0.024*
Immediate loading protocol	-0.494	0.262	0.61 (0.37-1.02)	3.56	0.059
Fixed full-arch prosthesis (all-on-4/6)	0.554	0.268	1.74 (1.03-2.94)	4.27	0.039*

Smoking status remained a significant predictor, with active smokers demonstrating a higher likelihood of receiving wide-diameter implants (adjusted OR = 1.88; 95% CI: 1.09-3.25; p = 0.024). Fixed full-arch prostheses were also independently associated with wide-diameter implants (adjusted OR = 1.74; 95% CI: 1.03-2.94; p = 0.039). Increasing age showed a modest but statistically significant association with wide-diameter implant selection, with each one-year increase in age increasing the odds by approximately 3% (adjusted OR = 1.03; 95% CI: 1.00-1.06; p = 0.046). Male sex (adjusted OR = 1.42; 95% CI: 0.85-2.38; p = 0.182), presence of systemic disease (adjusted OR = 1.56; 95% CI: 0.89-2.73; p = 0.119), and immediate loading protocol (adjusted OR = 0.61; 95% CI: 0.37-1.02; p = 0.059) were not significant independent predictors in the final model. The final GEE model demonstrated a good fit, with a QIC value of 218.4 and an overall classification accuracy of 76.4% (Table 4).

## Discussion

The present retrospective study evaluated implant selection patterns in full-mouth rehabilitation and identified factors independently associated with the selection of wide-diameter implants. The findings indicate that wide-diameter implants were used in 17.2% of the rehabilitated arches, and their selection was significantly influenced by patient-related anatomical, biomechanical, and prosthetic considerations. Specifically, bone augmentation, poor bone quality, posterior implant location, mandibular arch placement, smoking status, fixed full-arch prosthetic design, and increasing age were significant predictors of wide-diameter implant selection in this study. These findings provide valuable insights into contemporary clinical decision-making for implant-supported full-mouth rehabilitation.

One of the most important findings of this study was the strong association between bone augmentation and the selection of wide-diameter implants. Arches requiring bone augmentation were more than three times as likely to receive wide-diameter implants. This finding may appear counterintuitive because augmentation procedures are generally performed to compensate for inadequate bone volume. However, clinicians often use augmentation procedures to create sufficient ridge dimensions that permit the placement of implants with greater diameters, thereby improving implant stability and load distribution [14]. Buser et al. [15] reported that adequate ridge augmentation facilitates the placement of wider implants, which may improve stress distribution and long-term prosthetic support in cases of a compromised ridge. Similarly, Chrcanovic et al. [16] emphasized that implant diameter selection is often influenced by the need to maximize bone-to-implant contact following augmentation procedures.

Poor bone quality (Types III and IV) was another major determinant in the selection of wide-diameter implants. Clinicians frequently prefer wider implants in areas of reduced bone density because an increased implant diameter enhances the available surface area for osseointegration and improves primary stability [17]. This finding is consistent with that of Javed and Romanos [18], who demonstrated that implant diameter is an important determinant of primary stability in low-quality bone. Similarly, Sreerama et al. [19] concluded that compromised bone density necessitates modifications to the implant design and dimensions to achieve predictable outcomes. Therefore, the increased use of wide-diameter implants in Types III and IV bones observed in the present study likely reflects an evidence-based clinical strategy aimed at compensating for reduced bone support.

The posterior region was significantly associated with the placement of wide-diameter implants. This finding is clinically relevant because the posterior segments are subjected to greater occlusal forces during mastication. Wider implants may reduce crestal stress concentrations and improve biomechanical load transfer in these regions [17]. Misch [20] reported that posterior implants are frequently selected with larger diameters to counteract increased functional loading and to reduce the risk of mechanical complications. Similar observations have been reported by Anitua et al. [21], who found that implant diameter is often increased in the posterior areas to improve the force distribution and enhance prosthetic stability.

Mandibular arches were also significantly more likely to receive wide-diameter implants than maxillary arches were. The mandible generally exhibits denser cortical bone and greater resistance to functional loading than the maxilla. Consequently, clinicians may be more inclined to use wider implants in mandibular rehabilitation, where adequate bone volume allows their placement [22]. This observation is consistent with previous reports indicating that mandibular implant sites frequently accommodate larger implant diameters, particularly in bone-augmented ridges [22,23].

Smoking was identified as an independent predictor of wide-diameter implants. Although smoking is traditionally associated with impaired healing and increased implant complications, clinicians may compensate for the perceived risk by selecting implants with larger diameters to maximize stability and osseointegration potential [24]. Nevertheless, the relationship between smoking and implant diameter selection has received limited direct investigation and warrants further exploration. The significant association between fixed full-arch prostheses and the selection of wide-diameter implants highlights the biomechanical considerations involved in complete-arch rehabilitation. Fixed full-arch restorations generate substantial functional loads, particularly in the distal cantilever regions. Wider implants may provide enhanced support and reduce stress concentrations at the implant-bone interface [25]. Malo et al. [26], in their seminal work on the all-on-4 concept, emphasized the importance of implant distribution and dimensions in achieving long-term prosthetic success. The present findings support the concept that clinicians preferentially select wider implants when planning fixed full-arch prostheses.

Increasing age was modestly but significantly associated with the selection of wide-diameter implants. Older patients often exhibit age-related reductions in bone quality and remodeling capacity [27]. Consequently, clinicians may favor wider implants to enhance primary stability and maximize bone-to-implant contact. Although the effect size was relatively small, this finding suggests that patient age remains a consideration during implant treatment planning. Interestingly, immediate loading lost statistical significance after adjustment for the multivariable GEE model. This finding suggests that the apparent association observed in the univariable analysis was likely influenced by confounding factors, including bone quality, implant location, and prosthetic design. The use of GEE enabled more accurate estimation by accounting for clustering of arches within patients, thereby strengthening the validity of the final model.

The clinical implications of the present study are substantial. Understanding the factors that influence implant selection can help clinicians optimize treatment planning, improve biomechanical performance, and enhance long-term rehabilitation outcomes. The results suggest that implant diameter selection is determined not by a single factor but by a combination of patient characteristics, anatomical conditions, and prosthetic requirements. Recognition of these relationships may facilitate more individualized and evidence-based implant therapies.

Despite its strengths, this study had several limitations. The retrospective design inherently limits the ability to establish causal relationships and is subject to selection and information biases. Data were obtained from existing clinical records; therefore, some potentially relevant variables, such as insertion torque values, implant stability quotient measurements, clinician experience, and specific implant system preferences, could not be evaluated. Additionally, the study was conducted at a single center, which may limit the generalizability of the findings to other populations and treatment settings. Long-term implant survival and prosthetic success outcomes were not assessed, preventing the direct evaluation of the clinical effectiveness of different implant selection strategies. Future multicenter prospective studies incorporating long-term follow-up and standardized implant protocols are recommended to validate the present findings and further clarify the determinants of implant selection in full-mouth rehabilitation.

## Conclusions

Within the limitations of this retrospective study, implant selection in full-mouth rehabilitation was influenced by a combination of anatomical, biological, and prosthetic factors. Bone augmentation, poor bone quality, posterior implant location, mandibular arch placement, smoking status, fixed full-arch prosthetic design, and increasing age were independently associated with the selection of wide-diameter implants. These findings suggest that implant diameter selection is primarily driven by clinical and biomechanical considerations aimed at optimizing stability and load distribution. Further prospective multicenter studies are required to validate these findings and assess their impact on long-term treatment outcomes.
